# Thrombotic Microangiopathy Due to Malignant Hypertension Treated Exclusively With Antihypertensive Therapy

**DOI:** 10.7759/cureus.21835

**Published:** 2022-02-02

**Authors:** Taro Asano, Hideki Mori

**Affiliations:** 1 Emergency Medicine, National Organization Nagasaki Medical Center, Omura, JPN; 2 General Internal Medicine, National Organization Nagasaki Medical Center, Omura, JPN

**Keywords:** clinical decision making, thrombocytopenia, adamts13 activity, platelet count, plasma exchange, antihypertensive therapy, malignant hypertension, thrombotic microangiopathy

## Abstract

Plasma exchange is the commonly considered therapy for the treatment of thrombotic microangiopathy (TMA); however, it is not always mandatory. We treated a patient who presented with malignant hypertension (MH) complicated by TMA using antihypertensive therapy that was not accompanied by plasma exchange. A 38-year-old woman with photophobia, diarrhea, fever, and severely elevated blood pressure was referred to our hospital. Blood test results revealed thrombocytopenia and hemolytic anemia, and ascites were observed on the computed tomography images. Although TMA was suspected, plasma exchange was not performed because the platelet count was not markedly low. Her blood cell counts improved after antihypertensive treatment, and she was discharged. The patient is currently under therapy and remains stable. Thus, TMA secondary to MH may improve using antihypertensive therapy, without the need for invasive plasma exchange. Considering the platelet count may be helpful in deciding whether plasma exchange is required.

## Introduction

Malignant hypertension (MH) is characterized by severe hypertension and hypertensive retinopathy that results in bilateral flame-shaped hemorrhages in the retina and papilledema [1,2]. In rare cases, patients with MH exhibit thrombotic microangiopathy (TMA) that develops because of mechanical stress on the red blood cells as they pass through narrowed arteries due to fibrinoid necrosis or edema caused by severe hypertension [2,3]. Although plasma exchange is the treatment of choice when TMA is suspected, recent reports indicate that immediate blood pressure control should be prioritized for TMA secondary to MH because of its pathophysiology, rendering plasma exchange unnecessary. However, making therapeutic decisions is difficult in clinical practice [3]. Platelet counts may help determine whether this procedure is warranted. We report a case of TMA secondary to MH that was successfully treated only with antihypertensive therapy.

## Case presentation

A 38-year-old woman with photophobia and gastrointestinal symptoms was referred to our hospital. She had persistent photophobia of the right eye for three months and recurrent diarrhea (approximately 10 times/day), which was followed by a feeling of abdominal fullness for two months. Three days before admission, she experienced the bilateral blurring of vision. The patient’s medical history was unremarkable; she neither had hypertension nor any known drug or food allergies. She was a non-smoker, consumed alcohol occasionally, and denied illicit drug use. She was unemployed and lived with her mother. Her parents had hypertension but no collagen or endocrine diseases. The patient generally appeared ill; she was febrile (38.2 ℃), severely hypertensive (238/138 mmHg), and tachycardic (114 bpm) but had no episodes of desaturation (98% on room air). The physical examination revealed a loud apical S2 heart sound, abdominal distention, and bipedal edema.

Blood test results indicated anemia (9.6 g/dL), thrombocytopenia (100,000 cells/μL), and reticulocytosis. The liver function test revealed high indirect bilirubin (2.1 mg/dL), high lactate dehydrogenase (624 U/L), and low haptoglobin (<10 mg/dL) levels. Schizocytes were observed on the peripheral blood smear, and the Coombs test result was negative. Creatinine level was elevated (2.48 mg/dL). The values of prothrombin time and international normalized ratio (1.03) were normal; however, there was a decline in the activated partial thromboplastin time (25.2 s). Levels of serum fibrinogen (266 mg/dL) and fibrin degradation products (5.7 μg/mL) were normal. A diagnosis of acute disseminated intravascular coagulation was excluded because there was no evidence of coagulation factor consumption or fibrinolysis. There were no clinical signs indicating rheumatic diseases, such as systemic lupus erythematosus, scleroderma, anti-phospholipid antibody syndrome, or vasculitis. Furthermore, tests for proteinase-3-anti-neutrophil cytoplasmic antibodies, myeloperoxidase anti-neutrophil cytoplasmic antibodies, antinuclear antibodies, and rheumatoid factors were negative, and there was no complement loss. Serum renin activity (>20 ng/mL/h) and aldosterone levels (326 pg/mL) were high.

**Figure 1:**
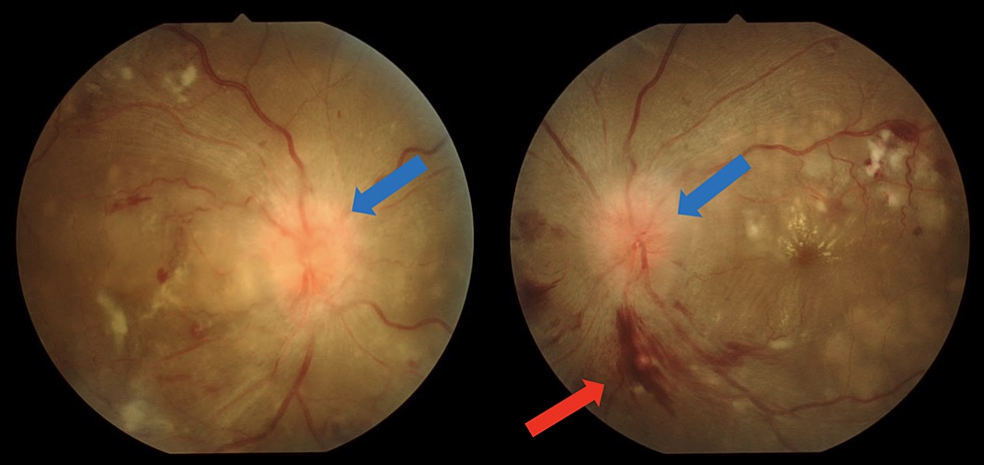
Fundus findings Bilateral flame-shaped hemorrhages (red arrow) and papilledema (blue arrows) suggestive of hypertensive retinopathy.

Verotoxin-producing Escherichia coli was absent; therefore, the hemolytic uremic syndrome was ruled out. Fundus findings revealed bilateral optic disc edema, retinal hemorrhage, retinal vitiligo, serous retinal detachment, and retinal vascular tortuosity, indicating hypertensive retinopathy (Figure 1).

Chest and abdominal computed tomography (CT) scans showed thickening of the left ventricle, enlargement of the ascending aorta, edema of the small intestine, peritoneal thickening, enlargement of the para-aortic and mesenteric lymph nodes, and ascites.

The patient was in a hypertensive emergency and admitted. Considering her untreated hypertension, platelet count of 100,000 cells/μL, and fundus findings that demonstrated hypertensive retinopathy and renal dysfunction, TMA due to MH was suspected. Although plasma exchange was considered to treat TMA, the platelet count was not low enough to deem it necessary. Intravenous antihypertensive treatment with calcium channel blockers and nitrites was initiated. On the next day, the platelet count improved, and the treatment was continued. On the fifth day of hospitalization, an ADAMTS13 activity of 38%, which did not decrease further, was detected.

**Figure 2:**
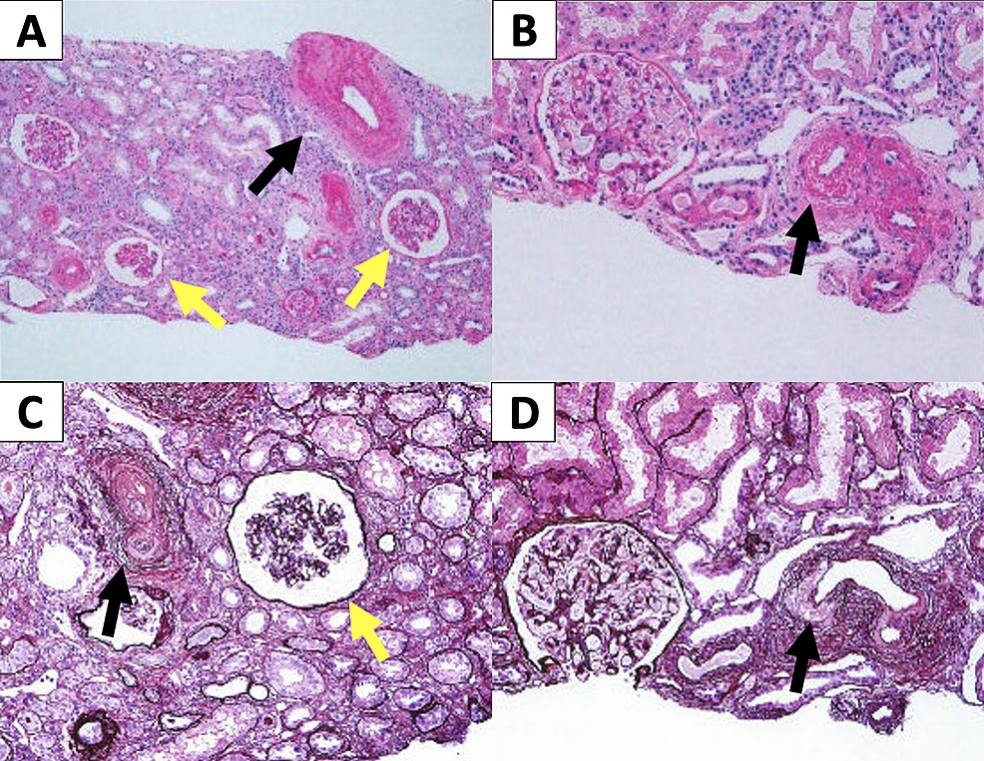
Renal biopsy Renal biopsy shows multiple sclerotic and collapsed glomeruli(yellow arrows), and lumen narrowing due to endothelial thickening with fibrosis in the interlobular arteries and arterioles(black arrows). There is no evidence for thrombosis in vasculatures such as glomerular tufts. A and B are Hematoxylin Eosin staining. C and D are Periodic acid-methenamine-silver staining.

Despite replacing the antihypertensive drugs with oral calcium channel blockers and angiotensin-converting enzyme inhibitors, the patient had elevated blood pressure during the night, and obstructive sleep apnea syndrome (OSAS) as a secondary cause of hypertension was considered. Sleep polysomnography was performed to confirm the diagnosis of severe OSAS, and continuous positive airway pressure therapy was initiated. Blood pressure and edema reduced, and after the patient was stabilized, renal biopsy was performed; it showed multiple sclerotic and collapsed glomeruli, narrowing of the lumen due to endothelial thickening, and fibrosis of the interlobular arteries and arterioles (Figure 2).

These findings confirmed TMA associated with chronic hypertension and MH.

The patient’s platelet count and general condition improved with antihypertensive therapy that was not accompanied by plasma exchange. Blood pressure was eventually controlled to within normal levels. She underwent rehabilitation and was discharged from the hospital. Currently, the patient continues therapy and remains stable.

## Discussion

The dysfunction of vascular endothelial cells in cases of MH is caused by activation of the renin-angiotensin-aldosterone system [4], which causes vasoconstriction that compensates for the release of vasodilators, such as nitric oxide, adrenomedullin, and prostacyclin from vascular endothelial cells. Prolonged hypertension reduces vasodilation and stimulates the secretion of proinflammatory cytokines by angiotensin II. Therefore, vascular endothelial cell damage and activation of the coagulation cascade leading to fibrinoid necrosis, arteriolar edema, and platelet aggregation. TMA is thought to be caused by mechanical stress when the erythrocytes pass through arteries that are narrowed because of fibrinoid necrosis and perivascular edema [2,3].

Owing to the pathophysiology of TMA induced by MH, rapid blood pressure control is essential, and treatment exclusively with antihypertensive therapy has been described in several case reports [5-8]. However, for treatment of hemolytic uremic syndrome (HUS)/thrombotic thrombocytopenic purpura (TTP) that is also related to MH, early initiation of plasma exchange should be considered because of the high mortality rate associated with it. Testing ADAMTS13 activity is considered useful in differentiating TMA due to MH from TTP/HUS of other etiologies; however, the long turnaround time of this test can cause delays.

Unnecessary plasma exchange therapy should be avoided because it is not only an expensive treatment but also associated with several complications, including allergenic reactions and catheter-related bloodstream infections. Thus, in clinical practice, a quick and simple method to distinguish between TMA due to MH and TTP/HUS from other causes is required. Platelet count can help predict decreased ADAMTS13 activity [9]. Shibagaki et al. reported that in cases of TMA secondary to severe hypertension, plasma exchange should be performed if the platelet count is less than 50,000 cells/μL even if the diagnosis is not confirmed. On the contrary, this therapy may be withheld in patients with severe or undiagnosed hypertension having a platelet count >50,000/μL that shows signs of improvement. Plasma exchange is performed later if the ADAMTS13 activity is low [2]. Thus, in addition to medical history and physical examination, paying particular attention to the platelet count may help avoid unnecessary plasma exchange therapy.

In this case, the patient presented with severe hypertension; however, her medical history, physical examination, and laboratory test results were not indicative of HUS/TTP because of other pathological conditions. Moreover, the platelet count was >50,000 cells/μL; therefore, the patient underwent only antihypertensive therapy. Consequently, the thrombocytopenia did not progress, and the patient recovered without plasma exchange therapy.

## Conclusions

TMA due to MH does not always require plasma exchange therapy and can be treated solely with antihypertensive therapy. However, early differentiation of TMA from TTP/HUS due to other etiologies may be difficult in clinical practice. Considering platelet count in addition to medical history and physical examination findings may help avoid treatment procedures.
